# Indents

**Published:** 1914-01-15

**Authors:** 


### INDENTS.

☞Brief Articles of Technical or Scientific Value will receive notice and comment. Contributions invited.

*New York Dentists May Not Prescribe Cocain.*

Druggists are not authorized to fill prescriptions calling for cocain when signed by dentists or veterinarians, according to a decision rendered yesterday by Attorney General Carmody at the request of the State Board of Pharmacy. The ruling is under the Walker cocain law, which was passed at the regular session of the legislature. In his opinion Mr. Carmody said in part:

"Sales may be made only to certain classes of persons, in the original packages, and in limited amounts. The classes to whom such sales may be made are pharmacists, druggists, including both manufacturers and dealers, physicians, veterinareans and dentists. Every sale must be recorded, with full details as to amount, date, and name of purchaser. All cocain purchased must be kept with two exceptions, in a place specified in the record of sale. The two exceptions as to keeping the drug in a specified place are on sale under physician's prescriptions and certain limited quantities which may be carried by a physician, veterinary or dentist for use in his profession.

"No provision is made for the filling of prescriptions by dentists or veterinarians, and such use of the drug as these two classes may make in their professions is therefore limited to that of direct, personal administration. An attempt by a dentist or veterinarian to use the drug by means of a prescription to be filled by a drgugist is penalized by making it a misdemeanor for any one, not of the classes specifically authorized, to have any of it in his possession without the certificate of the person making the sale, stating the name and address of the physician upon whose prescription the sale is made.

"I am, therefore, of the opinion that a druggist is not authorized to fill a prescription calling for cocain signed by a dentist or veterinarian, and that the use of the drug by dentists and veterinarians is limited to its purchase in original packages and direct administration to the patient."—*Albany* (N. Y.), *Press.*

*Sterilization of the Mouth.* Pane and D'Agata state that three years of further research have confirmed their previous statements in regard to the sterilizing action on the mouth of a 1 per cent. hot solution of sodium oleate. They describe research with the microbian flora of the mouth, the results showing how it is modified by this disinfectant which yet is free from irritating processes. To remove the taste of the soapy water they rinse the mouth out afterwards with some ordinary disinfectant, such as a 5 per cent. solution of potassium chlorate. They think that the stimulating effect of the latter hastens the cure.—*Journal American Medical Association.*

*Narcosis and Lack of Oxygen.* In a recent discussion of the nature and underlying factors of anesthesia[1], reference was made to one of the current views, championed particularly by Verworn, which maintains that a lowered oxidative function of the tissues is responsible for the anesthetic manifestations. On this hypothesis anesthesia would practically be equivalent to a sort of asphyxiation of the cells under the influence of the narcotic. Various telling objections have been offered in criticism of this point of view. It is true that there may be an intimate relation at times between the appearance of narcosis and a tendency toward deficient oxygen-supply in the tissues; but the two factors are by no means interdependent. Narcosis cannot be explained simply on the basis of an inhibition of oxidation, inasmuch as the possibility of narcotizing an individual is not inevitably associated with the existence of

1. A View of Anesthesia, editorial, THE JOURNAL A. M. A., Sept. 20, 1913, p. 968.

oxidative changes in the organism involved. This has now been demonstrated in a convincing way in the case of animals which exist quite independent of respired oxygen, namely, the anoxybiotic intestinal parasites. It is quite easy to keep the familiar intestinal roundworms, ascarides, alive and active in unoxygenated fluids, and still to narcotize them with alcohol or chloroform. They can thereupon be revived again by transfer into fresh fluid mediums without the participation of added oxygen at any time. The inhibition of oxidation in the higher forms of life must therefore at best be only an incident in the genesis of narcosis, not the fundamental determining feature.—*Journal A. M. A.*

*Analgesia and Analgesics on the Wane—Come now Vaccines.*

Men, like dogs, have fits; dentists are men, therefore dentists have fits. The analgesic fit in its more violent form is fading away, and like "the commercial side of dentistry" and "the management of a practice" will soon be regarded as one of those things that the profession "just had to do." "Fits" seem to be essential to progress; no fits, no progress. And it is well. The majority of us have had our turn with analgesics—rapid and rhythmic breathing, after the method of Bonwill; compression of the tissues from the outside; the same from within by injection of plain or salted water; the benumbing effects of the essential oils and narcotics superficially applied; chilling with rhigolene, ether and ethylchlorid; the injection of cocain or its equivalent into the tissues direct; and finally, the induction of analgesia by partial anesthesia by means of a general anesthetic "controlled" by oxygen.

If one may visualize along prosthetic lines the most conspicuous things on the professional horrizon at present are vaccines, the technique of production, their application in daily practice, reference being had particularly to the treatment of pyorrhea. Hardly a city of any size in the country is without its more or less enthusiastic experimentor in this field, and not a few of them have qualified to the degree that they speak with authority. Some of these are advocating the treatment in private practice and are making use of it; others are more cautious, feeling their way as one in the dark. Something bad will come of it of course; also a lot of good even if expectations are but half realized, for it does put the organism temporarily at least in the way of destroying or rendering inert a variety of toxic products.

This fit, again like the dog, will have its day, and when equilibrium has been established, it will be found to have added materially and substantially to our knowledge of one of the most troublesome, costly and least understood affections known to an ambitious and spasmodically active professional body.—*Dental Dispensary Record.*

*Tooth Conservation Injudicious.* It may be a triumph of skill to mount a serviceable crown upon a badly broken down root, restored to a healthy condition by well-directed treatment, but is it always judicious? Bifurcated roots, separately capped and the caps united, may make a firm foundation for a crown or a bridge abutment. It may be accepted as evidence of the operator's skill and it may delight the patient, but, can such an operation be kept clean? Will there not always be a space under the united caps that will prove in a short time a receptacle for much that should not be retained in a human mouth, and a possible menace to the patient's health? True such operations have done acceptable service at times for years, and have seemed to help out as nothing else could a defective denture. They are costly; they are so much more satisfactory than any other kind of restoration possible after their extraction that, after being done, one is loath to disturb them, and they are often retained long after their presence is accompanied by a foul odor indicating that all is not well. An unclean mouth is not a healthy mouth, and healthy conservers now fully recognize that uncleanliness may not cause present discomfort and yet be the direct cause of serious and irremediable trouble later on. The question is not, What can be done? but, What should be done? Any tooth or tooth root that cannot be rendered healthy with a prospect of remaining so for a reasonable time should be promptly extracted. All operations that invite unhealthy conditions a really skillful dentist will leave undone. This phase of our work calls for a long range "thinking cap." Hesitancy in forcep using is commendable, but promptness when promptness is called for is more so. One may live longer and enjoy life more with an "unfurnished" mouth than with a full complement of pus producers. —Dr. Trueman, in *Brief.*

*Relieving Undue Pressure of a Denture.* Now and again it is difficult to locate the exact spot on a denture that is causing an abrasion of the mucous membrane. In these cases place carefully over the abraded surface a piece of gummed paper, the gum side up. Moisten the denture and carefully place it in position with slight pressure. On carefully removing it the paper will be found adherent upon the point causing the trouble. —*Brief.*

*Fixing the Fee.* The Following articles (clipped from Dental Brief), "Business Nonsense" and "Fixing the Fee Problem," by Wm. H. Trueman, give food for serious thought, which the right-thinking dentist should absorb to his advantage:

Business Nonsense.—Many of the dental journals just now are full of business talk, very encouraging in the reading. One likes the idea of setting one's own salary and putting it at a liberal figure; the idea of increasing one's business by charging larger fees, increasing the income by working fewer hours, and making patients "stand around and pony up," is enchanting. It is like fighting battles while comfortably seated at one's fireside and no enemy in sight. It will likely come true when dentists let the supply houses send them supplies at will, instead of to order; the grocer and butcher the same, and the dentist insists in their accepting checks for twice the amount of the bills rendered. The business side of dentistry is the same as the business side of all other callings. The new idea may do for the men "high up"—but even to them it invites a fall—W. H. T.

Fixing the Fee Problem.—Fixing the fee problem is easily solved if all that is needed is to teach the patient the value of the service rendered, but it is quite another matter if the bread-winner of the family is one who earns ten or fifteen dollars a week only and is too proud to seek or accept charity. We commend that disposition in a humble wage-earner, and most of those who cater to his needs, the house builder, the tailor, shoemaker and grocer, those who provide him with literature and with amusements, thrive, while providing in full measure for his comfort and pleasure. One can get quite as much information from a volume costing eight or ten cents as from the same publication elaborately bound and costing dollars. There is a wide margin between comforts and luxuries when cost is considered. Tooth-saving dentistry is a necessity, and should be so considered when the relation of the dental profession to the community is under discussion. It is rather tantalizing to teach the general public the importance of saving the teeth by dental services and make the service so expensive that it is beyond the financial means of by far the largest number in an average community. He who can make two tooth-saving operations cost no more than one, and so enlarge the field and usefulness of the dental profession, is a much-needed philanthropist in every community A ten cent tintype gives as much satisfaction and serves as useful a purpose to thousands as does the oil portrait made by a master artist at a cost equal to a lifetime's earnings of a "hewer of wood and drawer of water," the tintype maker and the master artist have each a place among the world's workers and are equally entitled to credit when serving their respective clients equally well. When discussing the fee problem, consider well the question "Is dentistry a necessity or a luxury?"—W. H. T.

The analogy, "One can get quite as much information from a volume costing ten cents as from the same publication elaborately bound and costing dollars," is well founded except for the element of time as a factor in determining valuation, and "Fixing the fee problem is easily solved if all that is needed is to teach the patient the value of the service rendered," is a truth which applies equally in administering to all patients, regardless of financial conditions.

Unlike the medical man who can determine upon only one course of action at a time in any case, the dentist usually has several ways in which a tooth can be saved, each having its degree of permanency and consequently its individual value as a service rendered.

The fault lies, then in assuming toward a patient the attitude of dictator, instead of advisor and insisting that only the higher class of operation be performed whether the patient wants it and can afford it or not.

Saving a tooth is an imperative necessity; whether it is saved for the time being or saved for years depends largely upon the quality of "salesmanship," if it may be called such, and the ability of the dentist to perform the selected operation.

It is true that there are many articles appearing in our journals that are as much conducive of "grafting tactics" as they are of sound business judgment based on common justice, and tend to cloud the plain duty of the dental profession toward humanity.

The economic value of dentistry in its many important phases, once fully realized by the laity, will bring more financial reward to the individual members of the profession and do more to establish and maintain just fees than all the "business talk" advocating high-handed independence that we could read or listen to.—*Northwestern Journal.*

*Aluminum Solder.* Melt together six parts of tin and one part of Bismuth until well mixed. If more hardness is needed add a little more Bismuth.—*British Dental Journal.*

*Dental Public Service.* The Illinois State Dental Society has a committee called the Public Service Commission, which seems to have a new and useful object. During the last two years it has had thirty-four papers contributed to its various local and district societies on Medico-Dental Subjects, such as the influence of diseases of the mouth and teeth on general health. They also invite physicians to attend meetings where papers on such subjects as Oral Sepsis, etc., are given and they are asked to participate on the discussions. They also send prominent lecturers to various towns and cities to give popular lectures to which all the dentists, doctors, clergymen, members of school boards, officers of benevolent organizations, city officials and other interested people are invited. This is a commendable work and one that might be well done in every state.

*Softening Inlay Impression Wax.* Insert several pins into a large flat cork that will fit the mouth of the glass or vessel containing water heated to 140 degrees. Warm the pins and stick the balls of wax on the pins and insert them into the hot water, the wax will keep of proper temper for easy manipulation—J. D. McMillan, in *Review.*

*Short Broaches.* Cut off the shaft of the broach to any desirable length and fuse sealing wax on to it to make a suitable handle to grip in finger manipulation, facilitates broaching Molar Canals.—Dr. A. C. Willman, in *Review.*

*Adapting Porcelain Crown with Silicate Cement.* Prepare the root as desired and grind crown to approximate fit and adjust metal post. Dry root and insert post and place a piece of tin foil over the root end and surrounding gum to keep out moisture. Mix cement and place it on the porcelain crown and root end, and place the crown in position and hold it steadily until the cement sets enough to remove the crown and post without disturbing their relations. Lay the crown aside until the cement has thoroughly set, when it may be polished and set with oxyphosphate cement.—Dr. A. M. Wilkes, in *Review*.

*Pumice in Pyorrhea Pockets.* I strongly agree with Dr. Hartzell in condemning the application of pumice in pyorrhea pockets. If used, however, it should be followed by an injection of hydrogen dioxid, or, what I consider still better, a ten per cent. solution of lactic acid and a saturated solution of sodium bicarbonate.—Dr. Hofheinz, in *Cosmos*.

*Case of Sepsis, Following Dental Treatment, with Fatal Termination.* The wife of a physician had several carious teeth filled. Several weeks afterward, pain was experienced in a molar, which, upon opening, showed purulent pulpitis. Though the root-canal was treated, yet ulcerous endocarditis and swelling of the liver and spleen supervened, the patient succumbing four months afterward to general sepsis induced by way of the small pus focus in the pulp.—Dr. His, *Zahnaerztliche Rundschau.*—*Cosmos.*

*Classification of Oral Sepsis.* The type of unhygienic mouth, then, which is really associated clinically with lowered vitality is shown to be not necessarily the mouth reeking with caries, but the mouth where caries, or any other contributing cause, induces local lesions of the soft tissues; and this condition, for want of a better term, is called "oral sepsis." It covers a multitude of mouth sins, and is ably explained by D. F. Colyer when he says:

The term "oral sepsis" is used not to denote a specific disease, but collectively to include all chronic inflammatory diseases about the mouth;—there occurs an increase in number and variety of organisms commonly found in the mouth, especially those of the pyogenic class. Under altered environment many of these organisms may undergo change in virulence in the direction of exaltation or attenuation—some of those which were non-pathogenic may become pathogenic. The catarrhal and suppurative products which result from bacterial activity undergo putrefactive change, and in this condition are constantly passing into the gastro-intestinal tract with enormous numbers of bacteria.

It is apparent that the term covers all inflammatory mouth processes, and it is to just this lack of definition that we object; if the term pyorrhea alveolaris stands for confusion in pathology, then oral sepsis is "confusion worse confounded."

We can now classify oral sepsis as follows:

(A) Tissue exudate and pus freely evacuated into the mouth from pyorrhea, fistulous dento-alveolar abscess, or from any freely draining surface; ingestion and absorption of pyogenic products via the gastro-intestinal tract.

(B) Tissue exudate and pus retained in the alveolus from chronic "blind" dento-alveolar abscess and peridental abscess; pressure absorption via the blood stream by metastasis.

(C) A combination of the foregoing in advanced pyorrhea and dento-alveolar abscess, or deep necrotic surgical areas, which only drain occasionally; pus retention below the level of drainage. Damage results by methods in A and B.—Dr. Grieves, in *Nov. Cosmos.*

*Has Traveled 350,000 Miles.* The *Chicago Journal* has estimated that Dr. L. P. Haskell in going back and forth daily between Hinsdale, Ill., where he lives, and Chicago, where he practices, has traveled 350,000 miles. This is equal to about fourteen times around the world, and we think establishes a record so far as dental commuters is concerned. And he is still active and sprightly.—*Review.*

